# Editors’ Book Table

**Published:** 1874-03

**Authors:** 


### Editors' Book Table.

NOTE. — All works reviewed in the columns of the CHICAGO MEDICAL JOURNAL may be found in the extensive stock of W. B. KEEN, COOKE & CO., whose catalogue of Medical Books will be sent to any address upon request.]

*Dunglison's Medical Dictionary.* A new edition, enlarged and thoroughly revised, by RICHARD J. DUNGLISON, M.D. Philadelphia: Henry C. Lea. 1874.

Dunglison's Medical Dictionary has been a source of national pride to American physicians during the lifetime of the majority of those now in the profession. It has been no small boast to be able to claim, as our own, one of the best, if not the very best work of the kind which has ever appeared in any language. It might almost be termed an encyclopædia of medical sciences. It would be entirely superfluous to attempt a criticism of what has been the *vade mecum* of our professional lives, and is a familiar friend to all. We shall, therefore, only say to those who have found the old editions indispensable, that this only can still less be spared from the study table. The additions made by the Editor of the present edition, in order to keep up with the recent advances in medical and collateral sciences—more than six thousand words—one hundred and sixty pages—constitute, in themselves, a lexicon of no mean proportions nor feeble merit.

Dr. Dunglison could not have chosen a field of labor more prolific in fruit, more universally beneficial in its results, and more honorable to himself, than the reconstruction and elaboration of this monument of the genius, the industry, and the scholarship of his distinguished father. H.

*Sphygmograph: its Physiological and Pathological Indications.* The Essay, to which was awarded the Stevens' Triennial Prize by the College of Physicians and Surgeons, New York, April, 1873 Two hundred and ninety illustrations. By EDGAR HOLDEN, A.M., M.D. Philadelphia: Lindsay & Blakiston. 1874.

The invention of the sphygmograph by Marey attracted the general attention of physiologists, both in America and in Europe; for to it all looked as to the agency by whose means the pathological significance of changes in the circulatory system were to be unfolded, and its practical application was expected by many to initiate a new era in physical diagnosis. That the instrument has failed to fulfill these expectations has been for a long time apparent, and it is gratifying to recognize in Dr. Holden's Essay an effort to explain the reason why, and to demonstrate that "the question of real moment does not relate to the practical utility of any given sphygmograph, nor yet of the sphygmograph in its best known signification, but to whether there is any deep meaning in the blood current of the accessible arteries of value in physiology, pathology, or therapeutics, which may be accurately ascertained and recorded."

Convinced that the unsatisfactory results hitherto attained through the instrumentality of the sphygmograph were due to erroneous modes of construction, Dr. Holden directed his attention to the accomplishment of such modifications in the mechanism of the instrument as should obviate the causes of previous ill success. Perceiving that the instrument hitherto constructed was capable of utilizing the *lifting* power of the artery only, he attempted to construct a new one, which should be capable of utilizing the *displacing* power of the artery likewise. That this power of displacement is much more readily recognizable in its manifestations than the lifting power alone, is shown by the increased amount of pressure applicable to the artery by Holden's than by Marey's, Austin's, or Sanderson's instrument, viz., 700 to 1100 instead of 100 to 200 grammes respectively. The author claims for his instrument the advantages of ready applicability, and a diminution of two-thirds in its cost.

The chapter on arterial tension will repay careful study—in view of the prevailing obscurity and indefiniteness in the application of the term by practical writers—and in it the great importance of a correct appreciation of this condition in its relations to "the equilibrium of pressure, so essential to health," is rendered apparent.

The Essay epitomizes the results of several thousand sphygmographic tracings taken from cases of disease, more especially of some portion of the circulatory, respiratory, or nervous mechanism. The observations bear the impress of good faith, and of a desire to exhibit, honestly and fairly, the capacity of the instruments as a means of diagnosis, and not to uphold any pet theory.

Dr. Holden has, moreover, not limited the scope of his observations to the analysis of pathological conditions alone, but has extended them to experiments upon the determination of the effects of certain drugs upon his own person. Cannabis indica, gelseminum, aconite, and quinine, were administered experimentally, and their effects upon the circulation carefully measured by the sphygmograph, the collateral effects upon the nervous system being noted coincidently.

Dr. Holden's Essay is entitled to more than ordinary perusal; it deserves special study. Should it meet with the consideration it deserves, it will have initiated a new era in the history of the sphygmograph, which may culminate in its utilization as a means of diagnosis to a degree as yet inconceivable.

The style of the work is polished and elegant, proclaiming its author not only an earnest worker, but an accomplished scholar.

The typographical excellencies of the book entitle Messrs. Lindsay & Blakiston to the sincere thanks of all students, whose *res angustæ domi* compel them to use the day for labor and the night for study. H.

*The Anatomists' Vade Mecum.* A System of Human Anatomy. By ERASMUS WILSON, F.R.S. Edited by GEORGE BUCHANAN, A.M., M.D., Surgeon and Lecturer on Clinical Surgery, at the Glasgow Royal Infirmary, Professor of Anatomy in Anderson's University, assisted by HENRY E. CLARK, M.R.C.S. Ninth edition. Philadelphia: Lindsay & Blakiston. 1873.

Criticism of a work which has reached its ninth edition would be attempted too late, were one able to find an excuse therefor in this excellent treatise. A casual inspection of the book presents much that is attractive, and a more careful examination confirms and strengthens these favorable impressions. Its small size is a great advantage, contrasting favorably with the imposing dimensions of many of the volumes which have recently issued from the press, and which are indebted for their dimensions as much to padding as to their essential matter. The condensation of such a vast amount of valuable matter into so small a compass, is not only evidence of the admirably terse and concise style of the language, but likewise a great boon to students forced to realize so constantly that "life is short and art is long."

The different subdivisions of the subject matter have been carefully revised, and the contents brought down, or rather up, to the state of anatomical knowledge at date; they are more than usually complete. This may be especially said of the chapters on the nervous system, and of those upon the organs of special sensation. In the former we should have been glad to see a variation, which a due regard to physiology seems by this time to demand, in the phraseology by which a nerve trunk is described; thus, for example, instead of "the ophthalmic nerve arises from the upper part of the Gasserian ganglion," etc., etc., describe it as terminating there after arising from various points of origin, at which it receives impressions to be transmitted to the brain. The closer the association of anatomy and physiology in our text books, the more rapid will be our progress in the acquisition of that accurate knowledge of both, upon which alone rational medicine may rest.

The work is profusely illustrated, and the wood cuts are good, many of them, and those of the best, new. Taken altogether, it is the most attractive, and will prove to be the most useful manual of special anatomy accessible to the English speaking student.

H.

*A Handbook of Medical and Surgical Reference.* By JNO. A. WYETH, M.D., Member of the New York Pathological Society, etc., etc. New York: William Wood & Co., 27 Great Jones street. 1873.

A little book, which cannot fail to be very useful under the circumstances for which the author designed it, liable, however, to the objection which may be urged against all works of similar character, *i. e.*, the encouragement of indolence and routinism. This objection, indeed, the author deprecates, in advance, in his preface.

Although the suggestions in the work are all taken from standard authorities, we must presume to question the value of some of them, which appear to have the sanction of prescript and antiquity alone; *i. e.*, on page 83: "Apoplexy. If the apoplexy be dependent on active congestion, and the heart act with abnormal power, if there is a strong throbbing, resisting pulse, and the patient be full and plethoric, *blood letting* is indicated. Nine or ten ounces may be drawn from the *external jugular vein.*"

Apoplexy designating hæmorrhage into the cerebral ventricles, a condition in which a quantity of blood has been already withdrawn from the circulation, and most immediately from the circulation of the brain, we fail to see how the condition can be corrected by the removal of more blood.

We would have been glad to see the credit for the discovery of "Bibron's Antidote" (page 85) for snake bite, given to Dr. Daniel Brainard, of Chicago, to whom it belongs, instead of the mythical Bibron, whose existence was long ago shown to have depended upon a typographical error. On page 110 the use of gradually increased doses of strychnia for the relief of chorea, is attributed to Dr. Hammond; the credit properly belongs to Trousseau. The book is excellent of its kind, although we do not like this kind of book. H.

BOOKS RECEIVED.

*Dunglison's Medical Dictionary*—Henry C. Lea.
*Clinical Researches in Electro-Surgery*—By JULIUS E. ROCKWELL, M.D., and GEO. M. BEARD, M.D. William Wood & Co.,N.Y.
*Lecture on Bright's Disease*—JOHNSON. Geo. P. Putnam's Sons, N. Y.
*Anatomists' Vade Mecum*—Wilson & Buchanan.
*The Sphygmograph*—EDGAR HOLDEN, M.D. Lindsay & Blakiston, Philadelphia.
*A Handbook of Medical and Surgical Reference*—WYETH. William Wood & Co., N. Y.
*Bellamy's Guide to Surgical Anatomy*—Henry C. Lea, Philadelphia.
*Barnes on Diseases of Women*—Henry C. Lea, Philadelphia.
*Diseases of Children*—MEIGS and PEPPER. Lindsay & Blakiston, Philadelphia.
*Sanitary Association during the Franco-German War*, 1870-71. Vol. 1. The American Ambulance. THOS. W. EVANS, M.D., D.D.S., Ph.D. London: Sampson, Low, Marston, Low & Searle, 1873.
*Physicians' Office Case, Record and Prescription Blank Book*—GEO. E. WALTON, M.D., Cincinnati, Ohio.
*Griffith's Universal Formulary*—MAISCH. Henry C. Lea, Philadelphia. 1874.
*Wythe's Dose Book*—Lindsay & Blakiston. 1874.
*Galvano-Therapeutics*—PRINCE. Lindsay & Blakiston. 1874.

JOURNALS RECEIVED.

*The Clinic*—Cincinnati, Jan. 10, 17, 24, 31, Feb. 7.
*The Medical Times*—Philadelphia, Jan. 10, 17, 24, 31, Feb. 7.
*The Pharmacist*—Chicago, Jan. and Feb.
*The Medical and Surgical Reporter*—Philadelphia, Jan. 17, 24, 31, Feb. 7.
*The Journal of Chemistry*—Boston, Jan. and Feb.
*The Detroit Review*—Jan. and Feb.
*The Eclectic Medical Journal*—Cincinnati, Jan.

*The Vermont Medical Journal*—Burlington, Jan. Vol. 1, No. 1.
*The Southern Medical Record*—Atlanta, Dec. 1873 and Jan. 1874.
*The Physician and Surgeon*—Baltimore, Jan.
*The Central Law Journal*—St. Louis, Jan. 8, 22, 29, and Feb. 5.
*The Druggists' Circular*—Jan. and Feb.
*The Buffalo Medical and Surgical Journal*—Dec. '73 and Jan. '74.
*The Indiana Journal of Medicine*—Indianapolis, Jan.
*The Medical Examiner*—Chicago, Jan. and Feb.
*The American Practitioner*—Louisville, Jan. and Feb., '74.
*The Chicago Journal of Nervous and Mental Disease.*
*The Half Yearly Abstract of the Medical Sciences*—STONE.
*The Kansas City Medical Journal*—Jan.
*The Braithwaite's Retrospect*—Jan., '74.
*The Canada Medical and Surgical Journal*—Montreal, Jan.
*The Medical News and Library*—Jan. and Feb.
*The Boston Medical and Surgical Journal*—Feb.
*The New York Medical Record*—Jan. 15 and Feb. 2, '74.
*The St. Louis Medical and Surgical Journal*—Feb.
*The American Journal of the Medical Sciences*—Jan., '74.
*The New York Medical Journal*—Feb.
*The Nashville Journal of Medicine and Surgery*—Jan.
*The Richmond and Louisville Medical and Surgical Journal*—Jan.
*The Canada Medical Record*—Montreal, Jan.
*The Practitioner*—London, Jan.
*The Lancet and Observer*—Cincinnati, Jan.
*The Pacific Medical and Surgical Journal*—Jan.
*The North-Western Medical and Surgical Journal*, Jan.
*The Atlanta Medical and Surgical Journal*—Jan.

PAMPHLETS RECEIVED.

*Galvano-Therapeutics*—PRINCE.

*Formulæ for Elixirs*—American Pharmaceutical Association. HANCOCK, J. F.

*Transmission of Syphilitic Contagion*—R. W. TAYLOR.

*Report on the Immigration Service*—By JNO. M. WOODWORTH, M.D., Supervising Surgeon of Marine Hospitals.

*Transactions of Indiana State Medical Society.*

*The Larynx the Source of Vowel Sounds*—By THOMAS BRAIN GUNNING, New York.

*Report of the Committee on the Yellow Fever Epidemic of* 1873 *at Shreveport, La.* 1874.

*Report of the Board of Managers of the Western Lunatic Asylum of Kentucky.*

*Transactions of the Illinois State Medical Society.*

*Transactions of the American Ophthalmological Society.*

